# Cardiovascular Implantable Electronic Device Infection and New Insights About Correlation Between Pro-inflammatory Markers and Heart Failure: A Systematic Literature Review and Meta-Analysis

**DOI:** 10.3389/fcvm.2021.602275

**Published:** 2021-05-03

**Authors:** Stefania Zerbo, Giulio Perrone, Clio Bilotta, Valeria Adelfio, Ginevra Malta, Pietro Di Pasquale, Emiliano Maresi, Antonina Argo

**Affiliations:** ^1^Department of Health Promotion, Mother and Child Care, Internal Medicine and Medical Specialties, Section of Legal Medicine, University of Palermo, Palermo, Italy; ^2^Department of Economics, Business and Statistics, University of Palermo, Palermo, Italy; ^3^Division of Cardiology, Paolo Borsellino, G.F. Ingrassia Hospital, Palermo, Italy

**Keywords:** microbiology, heart failure, inflammatory markers, risk factors, CIED infection

## Abstract

**Introduction:** Surgical approaches to treat patients with abnormal pro-inflammatory parameters remain controversial, and the debate on the correlation between hematological parameter alteration in cardiac implantable electronic device (CIED) infection and the increase in mortality continues.

**Methods:** We performed a systematic review using the PubMed, Scopus, and Cochrane Library databases. Twenty-two articles from May 2007 to April 2020 were selected and divided according to the following topics: prevalence of microbes in patients with CIED infection; characteristics of patients with CIED infection; comparison between patients who underwent and did not undergo replantation after device extraction; and correlation between alteration of hematological parameters and poor prognosis analysis.

**Results:** Epidemiological analysis confirmed high prevalence of male sex, staphylococcal infection, and coagulase-negative staphylococci (CoNS). The most common comorbidity was heart failure. Complete removal of CIED and antimicrobial therapy combination are the gold standard. CIED replacement was associated with higher survival. High preoperative white blood cell count and C-reactive protein levels increased the risk of right ventricular failure (RVF) development. Increased red blood cell distribution width (RDW) value or decreased platelet count was correlated with poor prognosis. No correlation was noted between preoperative leukocytosis and CIED infection.

**Discussion:** A relevant correlation between leukocytosis and RVF was observed. Heart failure may be related to high RDW values and decreased platelet count. Data on the correlation between hematological parameter alteration and poor prognosis are missing in many studies because of delayed implantation in patients showing signs of infection.

## Introduction

An increase in the average age of the population, secondary to an improved quality of life and an evolution in the diagnostic and therapeutic approaches, caused a significant rise in the prevalence of heart diseases and placement of cardiac implantable electronic devices (CIEDs). CIEDs allow the restoration and control of the heart rhythm, ensuring high survival, especially in elderly subjects (1, 2).

Despite the benefits, the implantation or replantation surgical procedure presents a high risk of infection; the risk of surgical site infection is ~7% (3), and the prevalence of cardiac device-related infections ranges from 0.0 to 3.2%. Surgical site infection could be due to saprophytic microorganisms, contact with contaminated surgical instruments, or poorly decontaminated health workers. CIED infection could cause sepsis and prolonged admissions, patient debilitation, additional risk of complications, and increased healthcare costs. Multiple microorganisms can infect the cardiac device implantation or replantation surgical site. Among them are gram-positive and gram-negative bacteria, anaerobic bacteria, and fungi; in addition, there are polymicrobial infections and infections of unknown etiology. The most common microorganism is *Staphylococcus aureus*, a saprophytic bacterium generally present in the nose (30%) and deep layers of the skin (20%) (4–7). This bacterium can penetrate deep skin layers to the CIED pocket, adhering to prosthetic material and organizing into an antibiotic multiresistant structure: biofilm (8).

If microorganisms find favorable conditions for growth and multiplication, CIED infection can rapidly develop. Alternatively, the infection could also remain latent for an extended period, reactivating secondary to a trigger event (late infection). In the latter case, pocket CIED infection could spread along the catheters and the venous system to the heart and other organs, or cause disseminated infections due to bacterial metastasis. In severe cases, the infection can lead to death in ~20 and 50% of patients within 1 and 3 years, respectively (9). Approximately 50% of staphylococcal infections are methicillin-resistant.

CIED infection can be considered a predictable avoidable complication, according to recent literature. Surgical approaches to treat patients with abnormal hematological parameters (such as inflammatory parameters) remain controversial. The abnormal hematological parameters, considered negative prognostic factors in cardiac device implantation, are leukocytosis, erythrocyte sedimentation rate (ESR), C-reactive protein, thrombocytopenia, erythrocytopenia, increased creatinine levels, and low hemoglobin count. Due to an insufficient number of studies, the debate in the correlation between the alteration of hematological parameters and CIED infection, consecutive treatment failure, and increased mortality continues (10, 11).

A significant percentage of CIED infections are negative on microbiological tests for pathogen detection. Various factors could cause this event, such as a previous antibiotic therapy, inadequate samples, inappropriate culture medium, insufficient incubation time, fastidious microorganisms, and particularly the presence of a biofilm structure that makes it impossible to isolate bacteria using traditional sampling methods.

Microorganisms are organized into a complex structure, known as biofilm, that presents various features making pathogen identification difficult (12, 13), such as differences in their phenotype with the formation of small slow-growing colonies, extracellular polysaccharide matrices facilitating cell communication and microbial persistence, inhibiting phagocytosis (14), and microbial adherence to prosthesis surface. In addition, blood cultures cannot often diagnose fungal infections.

Therefore, microbiology investigation of purulent material from pocket CIED and postmortem examination is a diagnostic gold standard in these cases.

An autopsy is also fundamental in the sudden cardiac death of a patient with CIED infection (15), allows the diagnosis of CIED malfunctions, and excludes other causes of death in case of fatal arrhythmia after device extraction (16, 17).

Our study combines information from recent publications regarding the role of hematological parameters' alteration as they pertain to the prognosis of CIED implantation in patients with increased pro-inflammatory markers. In particular, this study aims to analyze patient characteristics, microbial population prevalence, replantation risk factors, and mortality outcomes for patients who are or are not replanted with CIED.

## Materials and Methods

In our study, two reviewers performed systematic electronic research of scientific articles on CIED infection and altered hematological parameters before CIED replantation. The articles were searched in the PubMed (MEDLINE), Scopus, and Cochrane Library (including the Cochrane Central Register of Controlled Trials, the Cochrane Database of Systematic Reviews) databases, and they were filtered by the following eligibility criteria: English language; types of study (clinical trial, meta-analysis, randomized controlled trial, review, systematic review), and human species. We selected the following combination of words: “CIED infection”; “Management implantable cardioverter-defibrillator infections”; “CIED pocket infection”; “Cardiac device related infection”; “Lead extraction without subsequent device implantation”; “Lead extraction after cardiac device implantation”; “Re-implantation after lead removal”; “CIED replantation”; “Indications replantation cardiac device”; “ICD lead extraction mortality”; “Replantation cardiac device extraction”; “TLE and cardiac device implantation”; “Leukocytosis and cardiac device implantation”; “Pre-operative pro-inflammatory response.”

We found 3,676 articles: 882 on PubMed, 2,578 on Scopus, and 216 on Cochrane Library.

**Figure 1:**
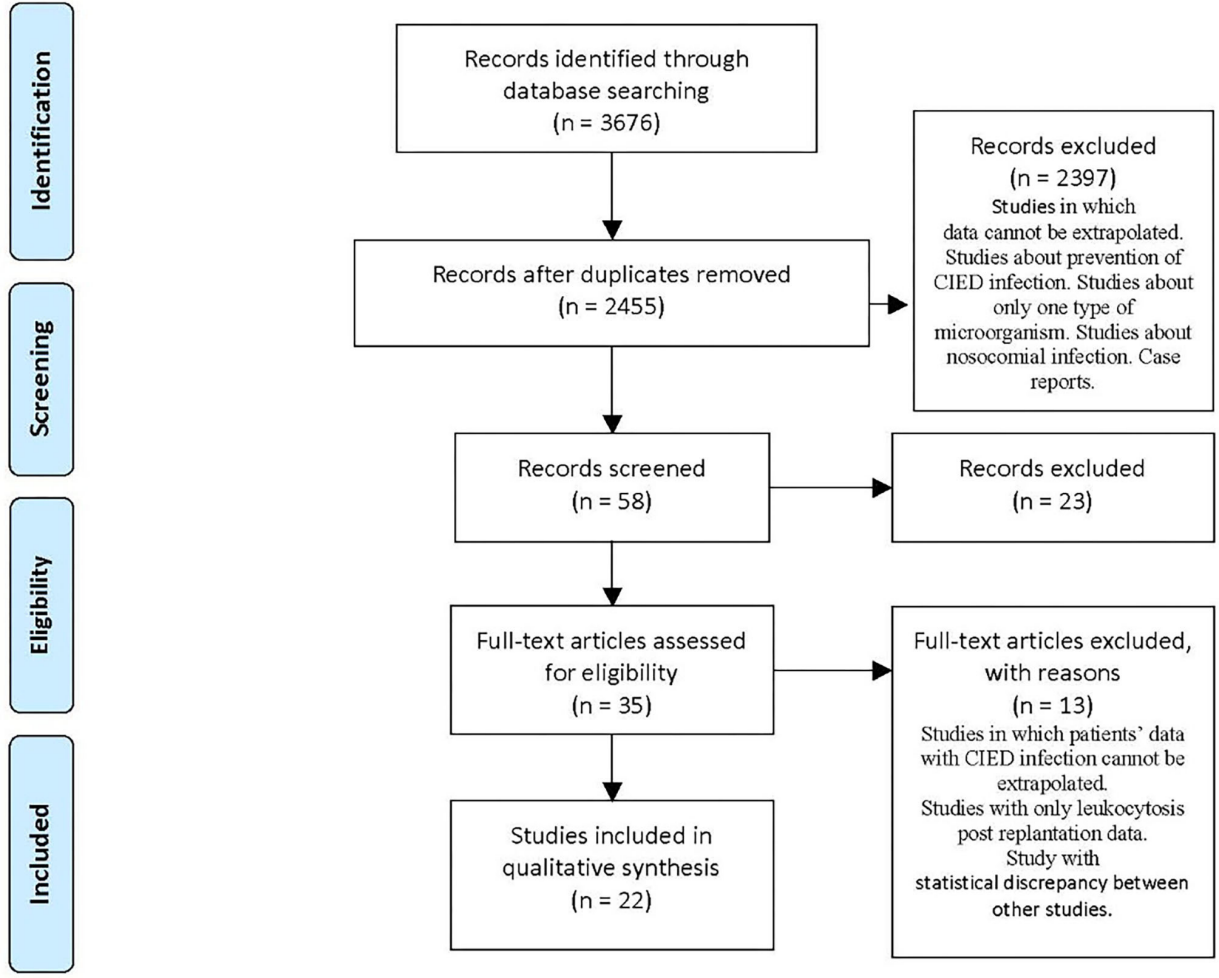
PRISMA flow diagram.

The remaining articles were 2,455 after de-duplication of 1,221 articles. We examined the titles and abstracts, and, where necessary, we opened articles in full text and analyzed them (Figure 1).

According to sequent inclusion criteria, we selected 22 articles from May 2007 to April 2020: titles and abstracts related to CIED and pro-inflammatory state prior to CIED replantation; full-text articles concerning microorganism prevalence data related to CIED infection; and articles inherent to the alteration of hematological parameters before replantation. Exclusion criteria consisted of studies with a single type of microorganism, languages other than English, leukocytosis post-replantation, case reports, and studies in which data of patients with CIED infection cannot be extrapolated.

We divided articles according to topics and organized them in Table 1, where we reported titles, authors, publication date, number of subjects, and study results. We applied significance tests for comparison of some subgroups.

**Table 1:** Analyzed studies.

**Study**	**Year**	**Type of study**	**Study** ** size**	**Proportion** ** of male**	**Prev. of** ** positive** ** response**	**Prev.** ** Staphylococcus** ** infection**	**Prev. CIED** ** complete removal** ** and antimicrobial** ** therapy**	**Prev. CIED** ** replacement**	**Mortality** ** rate**	**Prev. of** ** replanting**	**Prev. of** ** leukocytosis**
Wang et al.	2017	Retrospective	219	0.735	0.662	0.493	–	0.626	0.009	–	–
Viola et al.	2010	Retrospective	80	0.675	–	–	0.9125	0.538	0.038	–	–
Sohail et al.	2007	Retrospective	189	0.783	0.931	0.709	0.942	0.672	0.053	–	–
Le et al.	2011	Retrospective	416	0.755	1.00	0.673	0.904	0.642	0.147	–	–
Esquer Garrigos et al.	2019	Retrospective	354	0.757	1.00	0.811	0.944	0.647	0.014	–	–
Fukunaga et al.	2017	Retrospective	208	0.692	0.788	0.611	1.00	1.00	–	–	–
Greenspon et al.	2012	Prospective	145	0.696	–	–	0.959	0.503	0.283	–	–
Korkerdsup et al.	2018	Retrospective	54	0.759	0.648	0.407	0.815	0.426	0.019	–	–
Refaat et al.	2019	Retrospective	22	0.863	0.954	0.5	1.00	0.364	0.045	–	–
Rodriguez et al.	2011	Retrospective	350	0.757	0.851	0.649	1.00	0.380	0.094	–	–
Döring et al.	2019	Prospective	302	0.781	–	–	1.00	0.632	0.427	0.632	–
Al-Hijji et al.	2016	Retrospective	678	0.650	–	–	1.00	0.857	0.146	0.856	–
Nishii	2019	Retrospective	107	0.785	–	–	1.00	0.757	0.196	–	–
Rickard et al.	2013	Retrospective	151	0.830	0.864	0.617	1.00	0.536	0.440	0.574	–
Durante-Mangoni et al.	2013	Case Series	82	0.768	0.926	0.768	–	–	–	–	–
Ihlemann et al.	2015	Retrospective	71	0.760	–	–	1.00	-	0.141	–	–
Boersma et al.	2016	Retrospective	75	0.746	–	–	1.00	-	0.067	–	–
Tang et al.	2019	Retrospective	489	0.783	–	–	–	0.303	0.303	–	0.259
Papageorgiou et al.	2019	Retrospective	612	0.721	–	–	–	–	–	–	–
Aleong et al.	2020	Retrospective	8,248	0.675	–	–	1.00	1.00	–	–	–
Bongiorni et al.	2012	Retrospective	1,204	–	0.887	0.734	–	–	–	–	–
Smit et al.	2010	Retrospective	91	0.703	0.934	0.648	–	–	–	–	–
Global measure	–	–	–	0.707	0.889	0.689	0.992	0.660	0.171	0.761	

*CIED, cardiovascular implantable electronic device; Prev., prevalence*.

### Statistical Analysis

We analyzed patients with CIED implantation characteristics; we investigated the percentage distribution of gender, positive culture, infection type, type of therapy, CIED replantation, the number and causes of deaths, and comorbidities.

Prevalence was the primary analyzed measure; the different prevalence rates were calculated, also obtaining related confidence intervals.

We compared the different measures of proportion/prevalence using the forest plot that allowed heterogeneity analyses between studies to see if the global measure would reliably summarize individual study measures. The funnel plot allowed analyzing the presence of publication bias. The funnel plot usually is not used for prevalence measures because an upside-down funnel shape should express an average value not existing for the prevalence. For this reason, the funnel plot analysis remains merely theoretical in our work.

We used the z-test for the proportion analysis of male subjects to know if it was different from 0.5.

We applied the *t*-test to compare the averages of two groups and analyze the statistical differences.

We applied two types of measures to analyze the association between two factors: the odds ratio (OR) for retrospective studies and the relative risk (RR) for prospective studies.

We used the test of inconsistency for heterogeneity, examining whether we might achieve a global measure study uniting measures of each study.

We used a model with fixed or variable effects to calculate the odds ratio (OR) in some studies. A model with fixed effect is usually used, giving a weight to each study in consideration of its sample size and calculating OR by giving more importance to studies with high weight. We applied a model with variable effects to contain high studies' heterogeneity (I∧2≥90%); it provides OR estimates with a very wide confidence interval and gives more importance to studies with low weight.

Having different final objectives, not all the studies had the data necessary to perform the analysis.

## Results

### Microbial Prevalence in Patients With CIED Infection

We analyzed 12 studies, considering etiology and various subgroup microbial prevalences in 3,270 infections related to CIED implantation. All studies were retrospective.

We classified the pathogens during analysis of data into eight categories, as illustrated in Table 2A. Staphylococcal species were the most prevalent class of microorganisms (69%); it consisted of two subtypes: *S. aureus* and CoNS (Table 2B). Wang et al. (18) identified a predominance of staphylococcal infections (49%), with a CoNS prevalence of 92% and *S. aureus* prevalence of 8% in a sample of 108 staphylococcal infections. Most studies report a higher prevalence of *S. aureus* than CoNS. This ratio between *S. aureus* and CoNS is reversed with a CoNS-predominant role in some European studies, such as Durante-Mangoni et al. (23).

**Table 2A:** CIED related infection.

**Articles**	***N*^**°**^ of** ** patients**	**Staphylococcal** ** infection only**	**Streptococcal** ** infection only**	**GNR only**	**Anaerobic** ** only**	**Polymicrobial**	**Other**	**Negative** ** response**	**Fungi**
Wang et al. (18)	219	108		20		5	10	74	2
Sohail et al. (19)	189	134		17		13	8	13	4
Smit et al. (20)	91	59	5	8			13	6	
Le et al. (21)	416	280		30			106		
Bongiorni et al. (22)	1,204	884		65	27		77	136	15
Durante-Mangoni et al. (23)	82	63	6	4	1			6	2
Esquer Garrigos et al. (24)	354	287	3	31	12		21		
Fukunaga et al. (25)	208	127		8	14		13	44	2
Korkerdsup et al. (26)	54	22		6		5	2	19	
Refaat et al. (27)	22	11		4		3	2	1	1
Rodriguez et al. (28)	350	227		28			32	52	11
Rickard et al. (29)	81	50		6	2	2	10	11	
All	3,270	2,252	14	227	56	28	294	362	37
Percentage		68.9%	0.4%	6.9%	1.7%	0.8%	9%	11%	1.1%

*GNR, gram negative rod*.

**Table 2B:** Staphylococcal infection subtypes.

**Articles**	***N*^**°**^ of** ** patients with** ** Staphylococcal**	**Staphylococcal infection** ** only Subtypes**	
		**CoNS**	***S. aureus***
Wang et al. (18)	108	99	9
Sohail et al. (19)	134	79	55
Smit et al. (20)	59	26	33
Le et al. (21)	280	157	123
Bongiorni et al. (22)	884	737	147
Durante-Mangoni et al. (23)	63	46	17
Esquer Garrigos et al. (24)	287	158	129
Fukunaga et al. (25)	127	64	63
Korkerdsup et al. (26)	22	12	10
Refaat et al. (27)	11	7	4
Rodriguez et al. (28)	227	101	126
Rickard et al. (29)	50	27	23
All	2,252	1,513	739
Percentage		67.2%	32.8%

*CoNS, coagulase negative Staphylococci. S. aureus, Staphylococcus aureus*.

Bongiorni et al.'s (22) Italian study highlighted that the most prevalent CoNS pathogen was *S. epidermidis*, common in human skin tissue; therefore, the authors point out an increased risk of contamination from CoNS compared to *S. aureus* infections: in summary, a single *S. aureus*-positive blood culture is sufficient for diagnosing CIED infections, unlike CoNS species. In an Italian study, Durante-Mangoni et al. highlighted the importance of (at least) two positive blood cultures for the same antibiotype to confirm CoNS infections. An American study confirmed the same data: the need for (at least) two positive blood cultures for bloodstream infection (BSI) diagnosis (24).

If patient characteristics comply with systemic inflammatory response syndrome (SIRS) criteria or present symptoms, like hypotension or elements suggesting an underlying BSI, a single positive blood culture is enough.

Staphylococcal infections play a predominant role in CIED infection; however, in European and American literature, staphylococci cause two-thirds of total CIED infections, as pointed out by Smit et al. (20) and Sohail et al. (19), underlining the importance of empirical antibiotic treatment, including staphylococcal coverage, prior to microbiological results.

In methicillin-sensitive *S. aureus* (MSSA), an infection is necessary to modify empirical antibiotic treatment (30), replacing vancomycin, initially used for broad-spectrum empirical treatment, with antistaphylococcal penicillins (21).

Non-staphylococcal species infections, ranging from 10 to 30%, are characterized by two particularities: wide variety of pathogens and later onset of infection than staphylococcal species (3–6 weeks vs. 2 years).

Non-staphylococcal infections have a low prevalence of false positives because of rare skin contamination and a low death rate (25).

In lead-associated endocarditis (LAE), a CIED infection subgroup, *S. aureus* is the most common species even in late infections (31).

The comparison between sensible and resistant subgroups showed that methicillin-resistant Staphylococci (MRSA-MRSE) is associated with high mortality.

Finally, the fungal infection subgroup is related to immunocompromised patients and is distinguished by high mortality; in this subgroup, the most common microorganism is *Candida* spp. (32).

**Figure 2:**
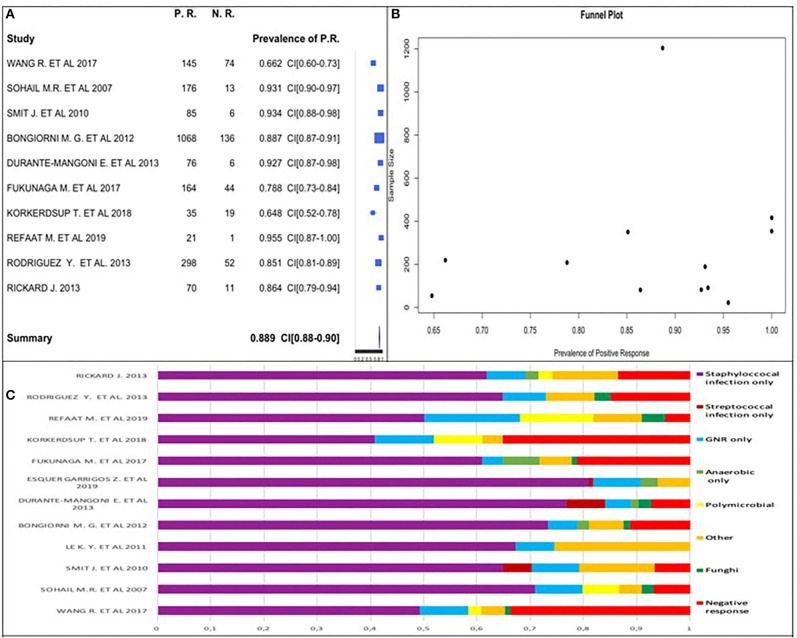
**(A)** Positive culture prevalence forest plot. **(B)** Positive culture funnel plot. **(C)** Percentage distribution of positive and negative cultures.

We used 10 (45.5%) of the 22 studies to calculate the positive culture prevalence (2 studies [9%] only reported on samples with positive cultures, and the remaining 10 [45.5%] did not report this information). The pooled prevalence estimate is shown in Figure 2A.

Two of the 12 excluded studies (7, 24) had all patients with positive culture (prevalence equal to 1); these two studies were included in positive culture global prevalence assessment. We observed that positive culture prevalence was between 64.8 and 95.5% in analyzed studies. Considering the studies' similarities, we obtained an overall prevalence estimate of 88.9%.

High positive culture prevalence was highlighted in patients with CIED infection.

We used a funnel plot (Figure 2B), including the two studies having prevalence equal to 1. It did not follow the upside-down funnel shape; we could not exclude the presence of publication bias. A correlation between the study sample size and a positive culture prevalence did not exist.

**Figure 3:**
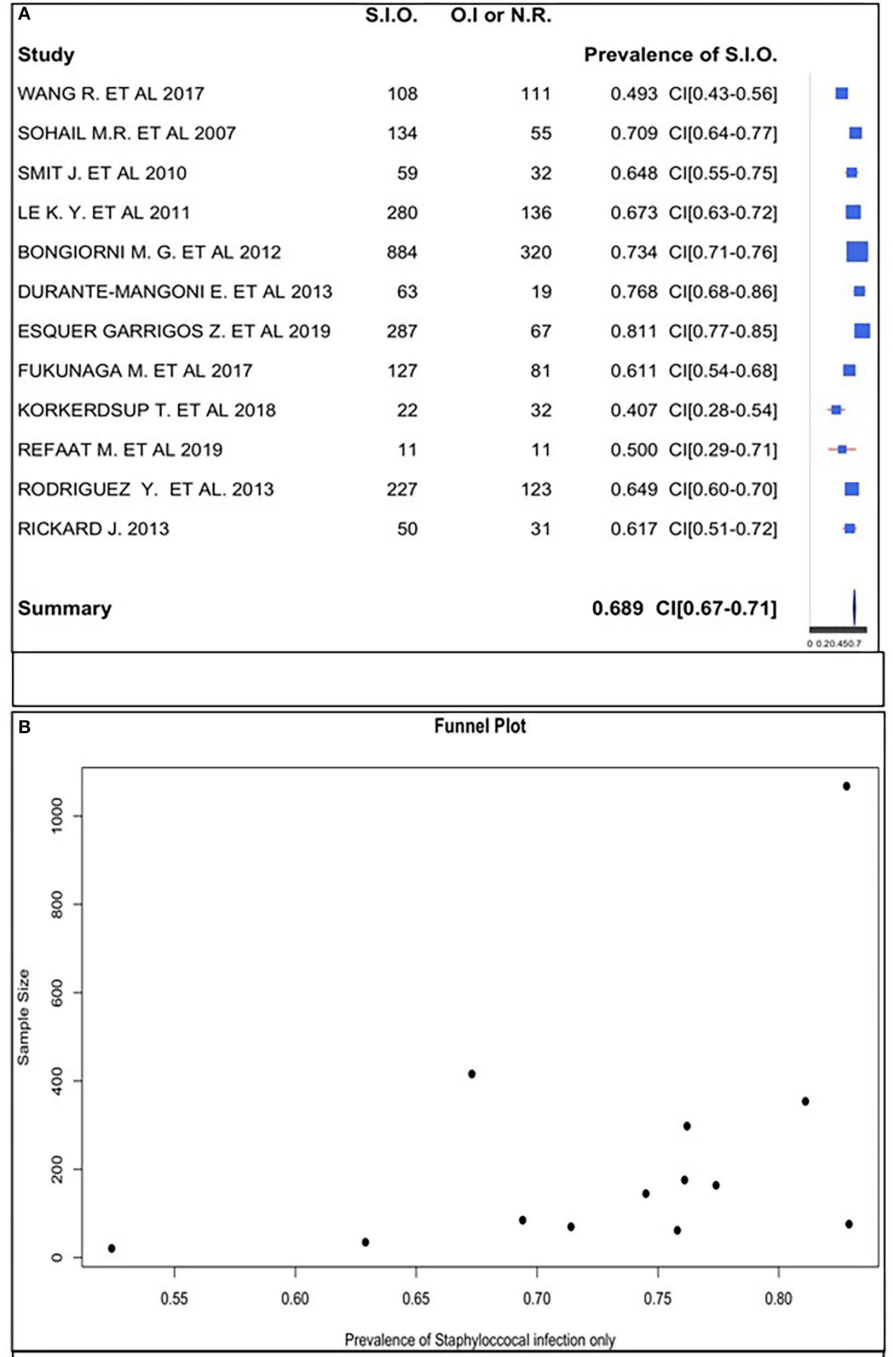
**(A)** Staphylococcal infection only prevalence forest plot. **(B)** Staphylococcal infection only prevalence funnel plot.

We estimated the prevalence of staphylococcal-only infection for studies with a positive culture to be 68.9%. The rates ranged from 40.7 to 81.1% across studies (Figure 3A).

We confirmed this datum analyzing 12 studies of 22 to calculate the positive and negative percentage distribution, as shown in Figure 2C.

The funnel plot did follow the shape of an upside-down funnel; thus, we could not exclude the presence of publication bias. The prevalence of only staphylococcal infection may increase with an increase in the sample size in the funnel plot (Figure 3B).

### CIED Infection Patients' Characteristics

We analyzed 19 articles with 11,895 cases of CIED infection. The patients' characteristics are described below.

#### Age and Gender

Regarding age, all studies reported the median patients' ages, except Aleong et al. (33). We calculated the mean of the 21 studies' median ages to be 69.15 years and the median to be 69.5 years. The lowest median age was 55.5 years (34); the highest median age was 73 years (25).

**Figure 4:**
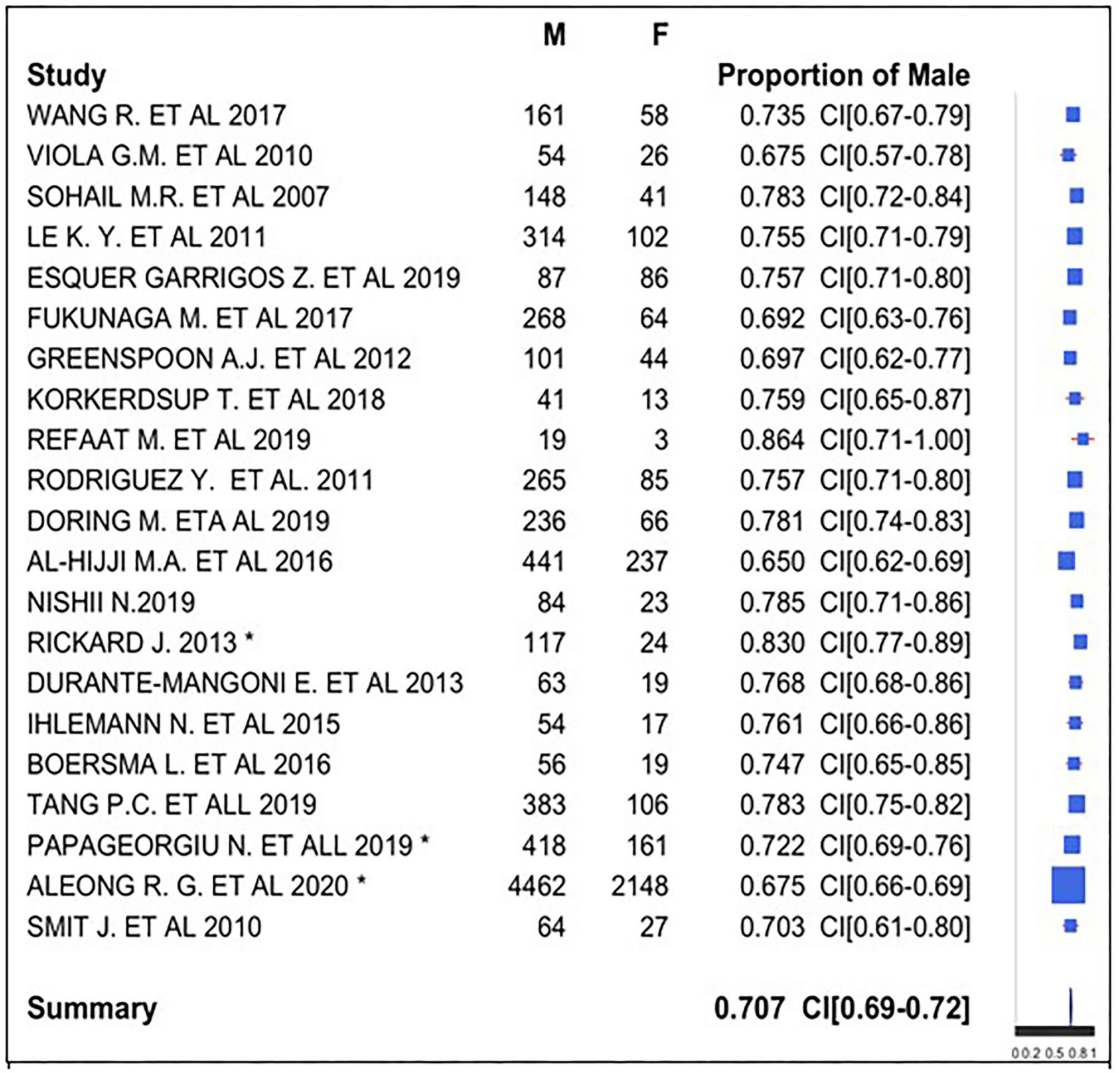
Gender ratio forest plot.

Regarding gender, we used a forest plot for male gender proportion (Figure 4). We analyzed this proportion in 21 of the 22 studies because 1 study (22) provided discordant data about gender, indicating a sample size (1,204) not corresponding to the sum of the male (939), and female (625) subjects.

The symbol “^*^” indicates the presence of unknown gender of subjects in the study: there were 10 subjects with unknown gender in Rickard et al. (29), 33 (5.4%) in Papageorgiou et al. (35), and 1,638 in Aleong et al. (33) (19.9%).

We registered male gender prevalence in all studies, with a proportion of 65–86.4%. We observed a similarity in the studies, and therefore, we obtained a global prevalence measure equal to 70.7%. We can conclude that there was a male gender prevalence in subjects with CIED. We used the z-test to assess whether the final proportion is statistically different from 0.5 (equal proportion between gender). The *P*-value was lower than the threshold value (0.05); data proportion was significantly different from 0.5. There was a prevalence of male gender in CIED implantation.

#### Comorbidities

The 18 selected articles described the following patients' comorbidities, as shown in Table 3: anemia, anticoagulation, cancer, cerebrovascular diseases, chronic kidney disease, chronic obstructive pulmonary disease (COPD), diabetes mellitus (DM), heart failure (HF), heart rhythm disorders, heart organic diseases, hypertension, immunosuppressive or corticosteroid therapy, ischemic cardiac diseases/coronaropathy, liver diseases, septic lung involvement, skin disorders, and valve diseases. The most common comorbidities consisted of cardiac diseases. The prevalence of HF was 46% of 10,242 cases analyzed in 12 articles: Viola et al. (36) reported an HF prevalence of 76% (61 patients); it was 51% (96 cases) in Sohail et al. (19); 41% (3,415 patients) in Aleong et al. (33); 58% (207 patients) in Esquer Garrigos et al. (24); 18% (37 patients) in Fukunaga et al. (25); 26% (37 patients) in Greenspon et al. (31); and 9% (5 patients) in Korkerdsup et al. (26). Refaat et al. (27) analyzed 22 patients with HF prevalence of 77%. All patients suffered from HF in Al-Hijji et al. (38) (678 patients) and Nishii et al. (39) (107 cases). HF prevalence was 38% (31 patients) among 82 patients in Durante-Mangoni et al. (23) and 89% (67 patients) among 75 patients in Boersma et al. (34).

**Table 3:** Comorbidities and treatment.

**Article**	**Wang** ** et al.** ** (18)**	**Viola** ** et al.** ** (36)**	**Sohail** ** et al.** ** (19)**	**Aleong** ** et al.** ** (33)**	**Le** ** et al.** ** (21)**	**Esquer** ** Garrigos** ** et al.** ** (24)**	**Fukunaga** ** et al.** ** (25)**	**Greenspon** ** et al.** ** (31)**	**Korkerdsup** ** et al.** ** (26)**	**Refaat** ** et al.** ** (27)**	**Rodriguez** ** et al.** ** (28)**	**Döring** ** et al.** ** (37)**	**Al-Hijji** ** et al.** ** (38)**	**Nishii** ** et al.** ** (39)**	**Rickard** ** et al.** ** (29)**	**Durante-Mangoni** ** et al.** ** (23)**	**Ihlemann** ** et al.** ** (40)**	**Boersma** ** et al.** ** (34)**	**All**	**Percentage**
**COMORBIDITIES**																				
Sample size	219	80	189	8,248	416	354	208	145	54	22	350	302	678	107	151	82	71	75	11,751	100%
Anemia				1,846															1,846	15.7%
ACT			67			122	89	34	2	7			425		80				826	7%
Cancer		11	24			38			1						13				87	0.7%
Stroke		11							6	1					18			6	42	0.3%
CKD	27	5				71	41	21	5	11	159	16	36	1	13	18	17		441	3.7%
COPD	23	5	34			48			4	2				19	18	15			168	1.4%
DM	53	23	46	2,221		90	30		16	8	177	141	175	30	60	25	12	22	3,129	26.6%
HF		61	96	3,415		207	37	37	5	17			678	107		31		67	4,758	40.5%
HRD		20	161	2,635	350				40	10		190	433		71			19	3,929	33.4%
OHD					4				8		7	61	384						464	3.9%
HT	106	70		3,206			120		29	15	304		309	56	78		22	37	4,352	37%
IT		6			43	36		14	2	1				5					107	0.9%
CAD		47	114				30	39	8	12	240	58	305		95		36	45	1,029	8.7%
Liver disease	24								1										25	0.2%
Septic lung involvement																31			31	0.2%
Skin disease						27													27	0.2%
Valve disease		7				55	10	18	1	6							12	10	119	1%
NA	219																		219	1.9%
**TREATMENT**																				
AT		5	4		21	20			8							NA			58	0.5%
AT+CS					1											NA			1	0.0%
CIED PR + AT		2	7		17			6	2							NA			34	0.2%
CIED CR + AT		73	178	8,248	376	334	208	139	44	22	350	302	678	107	151	NA	71	75	11,356	96.6%
CIED replacement	137	43	127	NA	267	229	NA	73	23	8	133	191	581	81	81	NA	71	75	2,120	18%

*ACT, anticoagulation therapy; CKD, chronic kidney disease; COPD, chronic obstructive pulmonary disease; DM, diabete mellitus; HF, heart failure; HRD, heart rytm disorders; OHD, organic heart disease; HT, hypertension; IT, immunosuppressive therapy; CAD, coronary artery disease; NA, not available; AT, antimicrobial therapy; CS, conservative surgery; PR, partial removal; CR, complete removal*.

Therefore, more than 50% of CIED infection patients suffered from HF in 7 of the 12 articles analyzed.

Hypertension was the second most common comorbidity: its prevalence was 42% in 12 articles analyzed (10,261 patients). More than 50% of patients suffer from hypertension disease in 7 of the 12 articles. The hypertension prevalence was particularly significant in two studies: 87.5% of 80 patients in Viola et al. (36) and 87% among 350 patients in Rodriguez et al. (28). We found a high hypertension prevalence in other studies: 68% of 22 patients in Refaat et al. (27); 58% of 208 cases in Fukunaga et al. (25); 52% among 151 cases in Rickard et al. (29); 54% of 54 patients in Korkerdsup et al. (26); and 52% among 107 patients in Nishii et al. (39).

Heart rhythm disorder prevalence was 38% in a sample of 10,215 analyzed cases (10 articles). More than 50% of patients had this pathological condition in six articles: 85% (161 patients) in Sohail et al. (19); 84% (350 patients) in Le et al. (21); 74% (40 patients) in Korkerdsup et al. (26); 64% (433 patients) in Al-Hijji et al. (38); 63% (190 patients) in Döring et al. (37); and 47% (71 patients) in Rickard et al. (29).

Finally, DM was a common non-cardiac comorbidity: its prevalence was 28% in a sample of 11,190 patients analyzed in 16 studies. The higher prevalence of this disease was highlighted by Rodriguez et al. (28), with a prevalence of 51% among 350 patients.

Comorbidity data were in an aggregate form because some subjects had more than one pathology in the studies. This explains why the distribution of comorbidities was obtained by the ratio of each disease and the total number of pathologies, not specifying patient multimorbidity, due to aggregation of these data.

**Figure 5:**
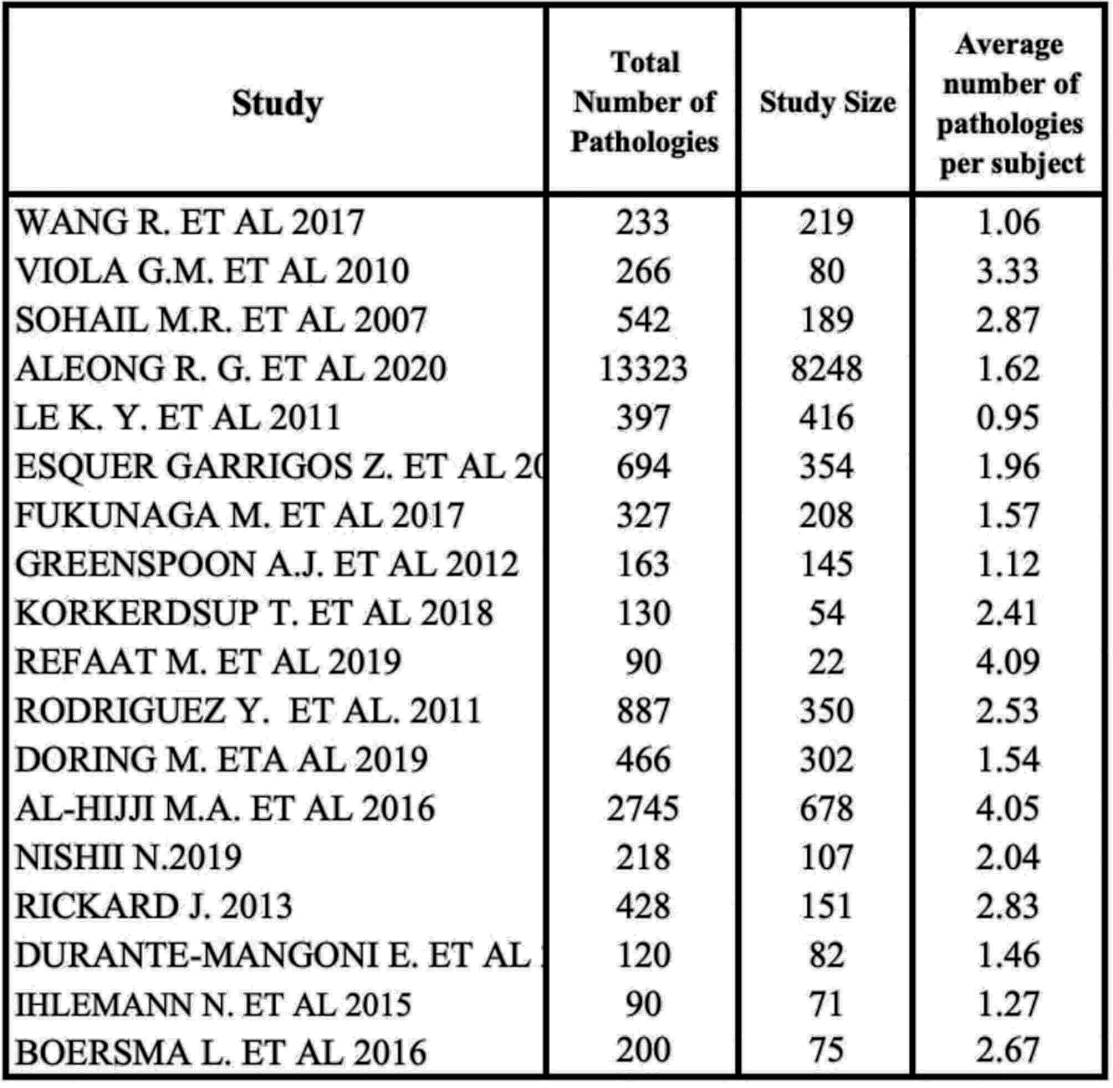
Average number of comorbidities for subject.

We analyzed the percentage distribution of comorbidities using 18 of 22 studies because of the absence of comorbidity data in 4 studies (20, 22, 35, 41). All studies outlined a high heterogeneity in the type and rate of diseases. The average number of pathologies per subject was achieved by a ratio between the total number of diseases and all patients' diseases of each study (Figure 5). A small subgroup with multiple co-pathologies or any pathology can influence this average, making it a non-standard measure. Refaat et al. (27) and Al-Hijji et al. (38) showed that the higher median number of pathologies per subject was equal to 4.

#### Treatment

Eighteen studies described patients' therapy in CIED infection in a sample of 11,751, as shown in Table 3. We found several treatment modalities: antimicrobial therapy alone; antimicrobial therapy and conservative surgery; CIED partial removal and antimicrobial therapy; and CIED complete removal and antimicrobial therapy.

CIED complete removal and antimicrobial therapy were the most commonly used options.

CIED complete removal and antimicrobial treatment combination therapies were applied to 100% patients in 10 studies: Aleong et al. (33); Fukunaga et al. (25); Refaat et al. (27); Rodriguez et al. (28); Döring et al. (37); Al-Hijji et al. (38); Nishii et al. (39); Rickard et al. (29); Ihlemann et al. (40); and Boersma et al. (34).

CIED complete removal and antimicrobial treatment was the most common chosen combination therapy in other studies: 91% of cases in a sample of 80 patients in Viola et al. (36); 94% cases of 189 and 354 patients, respectively, in Sohail et al. (19) and Esquer Garrigos et al. (24); 96% cases among 145 patients in Greenspon et al. (31); 90% of 416 patients in Le et al. (21); and 81% of 54 patients in Korkerdsup et al. (26).

CIED partial removal and antimicrobial treatment combination therapy was applied to 4% of patients in a sample of 884 patients in five studies (19, 21, 26, 31, 36).

CIED replacement was another considered data analyzed in all 15 studies. Its prevalence was 66% in a sample of 3,213 subjects. All patients were replanted in two studies (34, 40).

The replantation prevalence rate was higher than 50% in 10 studies: 86% of 678 patients in Al-Hijjii et al. (38); 76% of 107 patients in Nishii et al. (39); 67% of 189 cases in Sohail et al. (19); 65% in a sample of 354 patients in Esquer Garrigos et al. (24); 64% in Le et al. (21) (416 patients); 62.5% among 219 patients in Wang et al. (18); and 54% in Viola et al. (36) (43 patients); and Rickard et al. (29) (151 patients).

Antimicrobial therapy alone was applied to 5% of patients in a sample of 1,093 cases analyzed in five articles (19, 21, 24, 26, 36).

**Figure 6:**
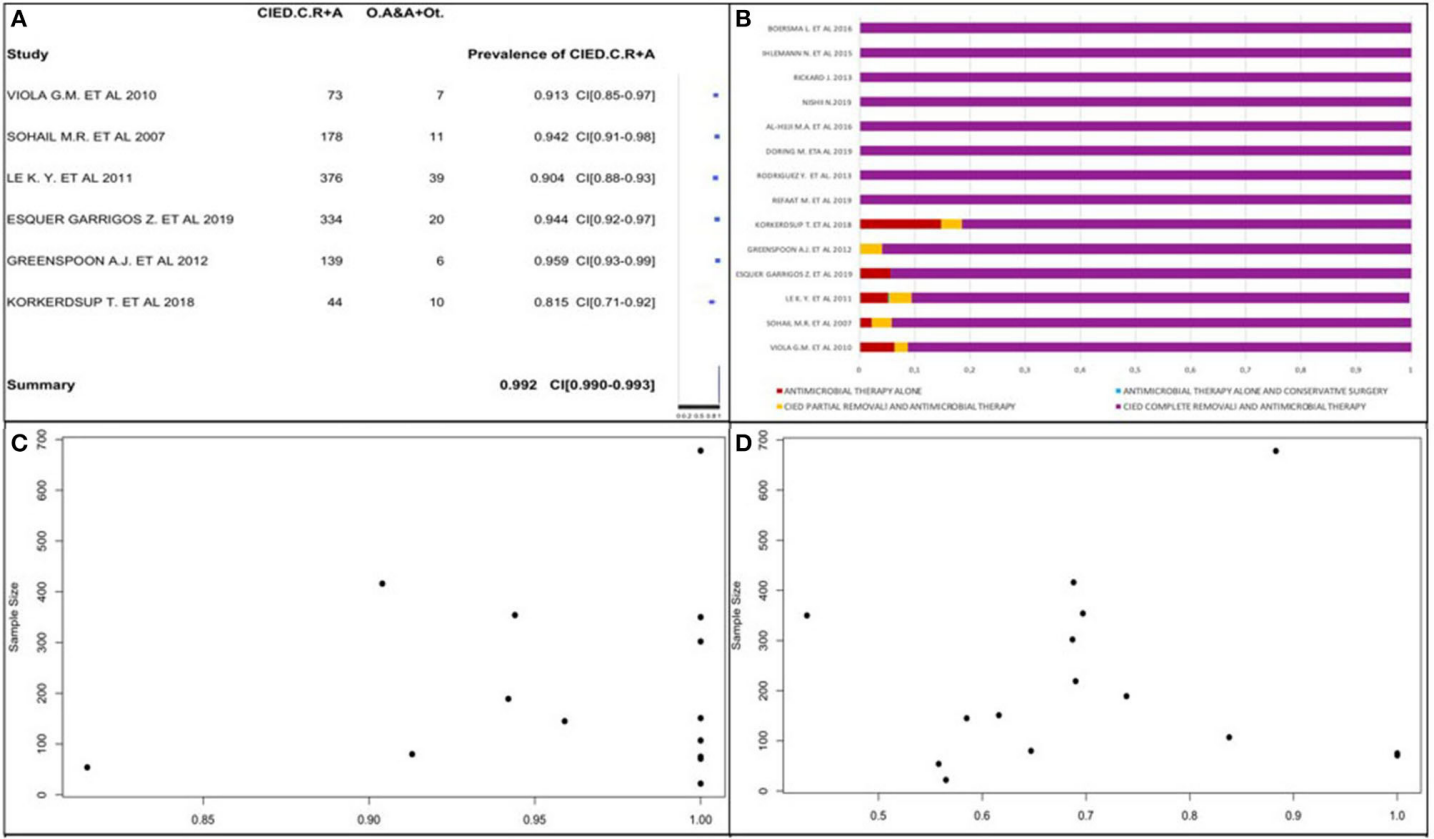
**(A)** CIED complete removal and antimicrobial therapy prevalence forest plot. **(B)** Post-operative therapy type percentage distribution. **(C)** CIED complete removal and antimicrobial therapy prevalence funnel plot. **(D)** Prevalence of replanted subjects funnel plot.

CIED complete removal and antimicrobial therapy was the prevalent treatment, so we obtained its prevalence using the forest plot (Figure 6A). We selected 16 of 22 studies because of the absences of data about post-operative treatment in the other 6 studies (18, 20, 22, 23, 35, 41). We used 6 of 16 studies because all patients received CIED complete removal and antimicrobial therapy in the other 10 studies (25, 27–29, 33, 34, 37–40). The prevalence of CIED complete removal and antimicrobial therapy was the most common. There was a similarity in the studies; thus, we obtained a global prevalence measure equal to 99.2%, being affected by the large sample size in Aleong et al. (33) (it represented 73.5% of the sample); this study increased the value only by 2%; therefore, it was included in the analysis. This post-operative approach was the most frequent, and it was often the only used therapy.

We confirmed these data by analyzing the percentage distribution of all post-operative therapies in each study, as shown in Figure 6B. It shows only staphylococcal infection prevalence.

We also obtained CIED complete removal and antimicrobial therapy prevalence using a funnel plot (Figure 6C), in which the study by Aleong et al. was not represented due to its large sample size. The plot did not surely allow reporting data about publication bias presence. There was no correlation between study sample sizes and post-operative therapy type prevalence.

We analyzed CIED replantation prevalence in 13 of 22 studies (Figure 6D) due to the absence of data in 7 studies and the exclusion of 2 others (34, 40) due to replantation of the whole population. The prevalence varied from 36.4 to 85.7%. It was not possible to obtain a publication bias and affirm that a high CIED replantation prevalence exists due to the studies' variability. We observed that most studies had a CIED replantation prevalence >50%. Using a funnel plot, we observed that it did not follow the shape of an upside-down funnel; we could not exclude publication bias.

#### Mortality and Death Cause

We analyzed 15 studies reporting the death rate as illustrated in Table 4. The authors reported the mortality rate according to different time related to recovery: in-hospital mortality, mortality within 1 month from recovery, after 3–6 months from recovery, and after 1 year from recovery.

**Table 4:** Mortality and causes of deaths.

**Article**		**Wang** ** et al.** ** (18)**	**Viola** ** et al.** ** (36)**	**Sohail** ** et al.** ** (19)**	**Le** ** et al.** ** (21)**	**Esquer Garrigos** ** et al.** ** (24)**	**Greenspon** ** et al.** ** (31)**	**Korkerdsup** ** et al.** ** (26)**	**Refaat** ** et al.** ** (27)**	**Rodriguez** ** et al.** ** (28)**	**Döring** ** et al.** ** (37)**	**Al-Hijji** ** et al.** ** (38)**	**Nishii** ** et al.** ** (39)**	**Rickard** ** et al.** ** (29)**	**Ihlemann** ** et al.** ** (40)**	**Boersma** ** et al.** ** (34)**	**All**	**Percentage**
	Sample size	219	80	189	416	354	145	54	22	350	302	678	107	151	71	75	3,213	
N° of deaths^*^	All	2	3	10	61	5	41	1	1	33	92	72	21	10	10	5	367	11.4%
	NA or UNK				61		40				92	44	3				240	7.4%
	Known	2	3	10		5	1	1	1	33		28	18	10	10	5	127	4%
Time of deaths	In-hospital	2	3	10		5		1	1	33				10			65	
	30 days				23												23	
	3–6 months						41										41	
	≥1 year				61						92	72	21		10	5	261	
Causes of deaths^**^	Sepsis	2	3	7					1	13		5		10			41	11.2%
	CVA											5					5	1.4%
	HRD											1				1	2	0.5%
	Cardiac death (unspecified)									16			4				20	5.4%
	CANCER											5			1		6	1.6%
	ESCM											3					3	0.8%
	ESRD											4			1		5	1.4%
	HF														5	3	8	2.2%
	HCAP							1							1		2	0.5%
	Intraoperative			3		5	1			2					2		13	3.5%
	IM											1					1	0.3%
	Non-cardiac death (unspecified)												14			1	15	4.1%
	PE									2							2	0.5%
	RF											3					3	0.8%
	Suicide											1					1	0.3%

*NA, not available; UNK, unknown; CVA, cerebrovascular accident; HRD, heart rythm disorders; ESCM, end-stage cardiomyopathy; ESRD, end-stage renal disease; HF, heart failure; HCAP, healthcare-associated pneumonia; MI, myocardial infarction; PE, pulmonary embolism; RF, respiratory failure*.

* *Number of deaths percentages were calculated by reference to sample size*.

** *Cause of deaths percentages were calculated by reference to number of all deaths*.

In-hospital mortality occurred in 18% among 367 total cases of death.

Le et al. developed the only study evaluating mortality within 1 month, with a prevalence of 38% in a sample of 61 dead patients.

Six studies (21, 34, 37–40) reported mortality within or more than a year with a prevalence of 16% among 1,649 patients.

We observed various cardiac and non-cardiac death causes: sepsis, cerebrovascular accident, arrhythmia, cancer, end-stage cardiomyopathy, end-stage renal disease, hemorrhage, hemothorax, heart failure, healthcare-associated pneumonia (HCAP), intraoperative, myocardial infarction (IM), pericardial complication, pulmonary embolism, respiratory failure, and suicide.

Two studies (28, 39) reported an unspecified cardiac death prevalence of 37% among 54 death patients; another two (34, 39) highlighted an unspecified non-cardiac death prevalence of 58% in 26 death patients.

We found 240 unknown or not available deaths in five studies (21, 31, 37–39). These deaths occurred with a prevalence of 64% in a sample of 367 death patients.

The most common cause of death is sepsis, reported with a prevalence of 31% in a sample of 131 death patients in seven studies. It resulted in all patients' death in four studies (18, 27, 29, 36).

Sepsis-related deaths account for 70% of all 10 deaths in the study by Sohail et al.

**Figure 7:**
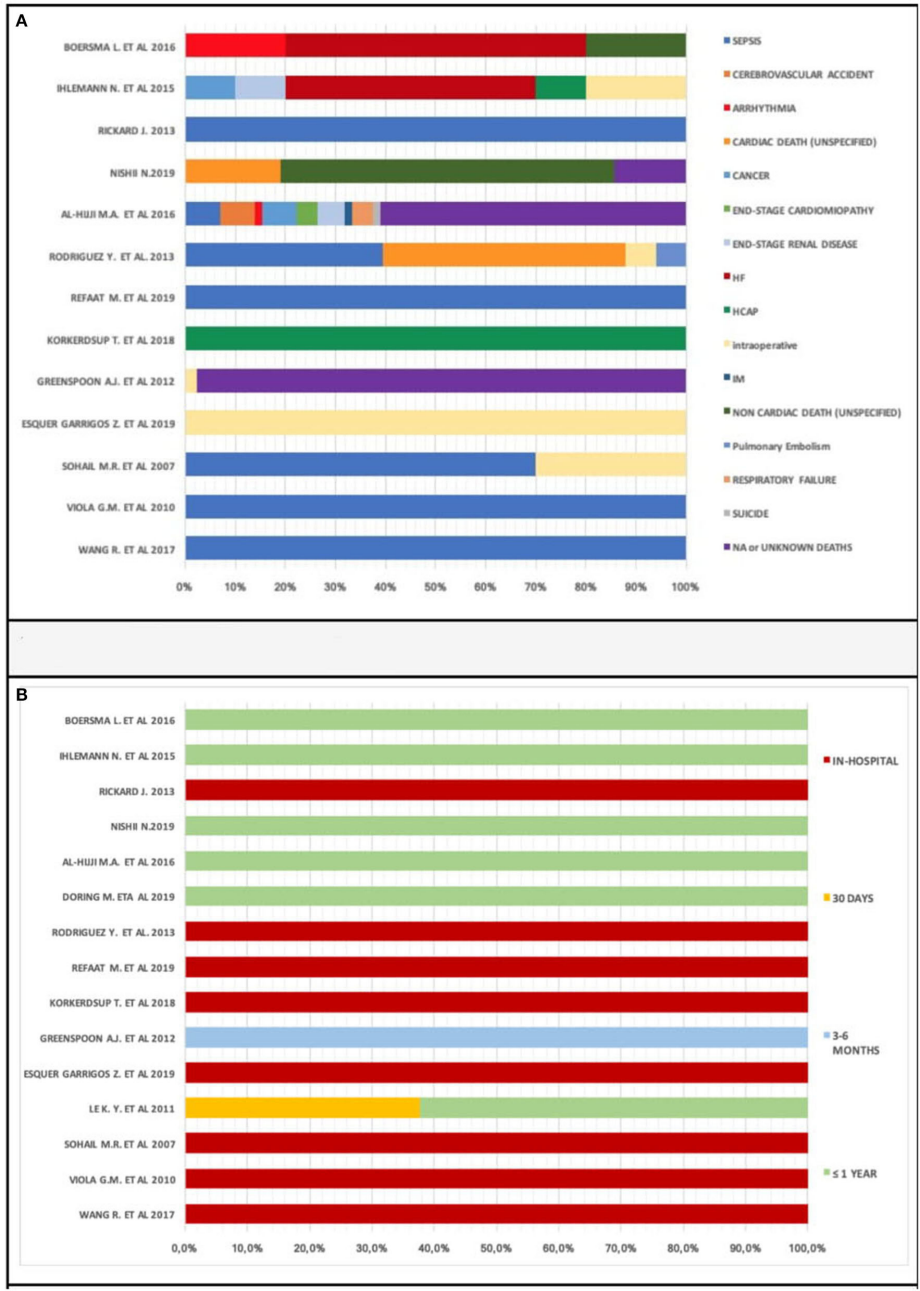
**(A)** Cause of deaths percentage distribution. **(B)** Times of deaths percentage distribution.

We analyzed causes of death percentage distribution in 13 of 22 studies (Figure 7A) due to the absence of data on the causes of death in 7 studies (20–23, 25, 33, 35, 37, 41). Sepsis was the most prevalent cause of death. However, there were various causes of death and lack of data; we cannot provide certainty for this reasoning. Different causes of death, therefore, contribute to a patient's demise after CIED implantation.

Times to deaths analysis showed early death prevalence (within 1 year): in-hospital subgroup percentage of deaths was 37.7% (Figure 7B). Only one study submitted data concerning within 1 month [(21) with a percentage of 37.7%] and 3–6 months mortality.

### Comparison Between Replanted and Patients Who Were Not Replanted After Device Extraction

In the analysis of three studies (Döring et al., Al-Hijji et al., and Rickard et al.), we compared two patient populations: replanted patients and patients who were not replanted after device extraction (Table 5). Overall, replanted patients were 853 (191 in Döring et al.; 581 in Al-Hijji et al.; 81 in Rickard et al.); patients who were not replanted consist of 268 (111 in Döring et al., 97 in Al-Hijji et al. and 60 in Rickard et al.). We examined the patients' characteristics in both groups.

**Table 5:** Patient characteristics according to replantation.

**Explanted and replanted patients**					**Percentage**	**Explanted and not replanted patients**			**Percentage**	***P*-value**
**Articles**		**Döring** ** et al. (37)**	**Al-Hijji** ** et al. (38)**	**Rickard** ** et al. (29)**		**Döring** ** et al. (37)**	**Al-Hijji** ** et al. (38)**	**Rickard** ** et al. (29)**		
Sample size		191	581	81		111	97	60		
Gender	M	159	374	73	71%	77	67	44	70.1%	0.838
	F	32	207	8	29%	34	30	16	29.9%	
Median age		70	70.6	71.4		73	60.7	66.4		
Comorbidities	AT	0	372	50	49.5%	0	53	30	31%	0.001
	CKD	8	24	3	4.1%	8	12	10	11.2%	0.019
	DM	89	168	31	33.8%	52	7	29	32.8%	0.837
	HF		581	0	68.1%		97	0	36.2%	0.002
	OHD	45	352	0	46.5%	16	32	0	17.9%	0.002
	HRD	112	400	43	65.1%	71	33	28	49.3%	0.012
	HT	0	276	47	37.9%	0	33	31	23.9%	0.024
	CAD	34	269	59	42.4%	24	36	36	35.8%	0.064

*M, male; F, female; AT, anticoagulation therapy; CKD, chronic kidney disease; DM, diabetes mellitus; HF, heart failure; OHD, organic heart disease; HRD, heart rhythm disorders; HT, hypertension; CAD, coronary artery disease*.

#### Age and Gender

The male sex had a higher prevalence in both groups: 71% of 853 replanted cases [83% of 191 patients in (37); 64% of 581 cases in (38); 90% of 81 patients in (29)] and 70% of 268 patients who were not replanted (69% of 111 cases in Döring et al.; 69% of 97 patients in Al-Hijji et al.; 73% of 60 cases in Rickard et al.). The average median age was 70.54 years for replanted patients (70 years in Döring et al.; 70.6 years in Al-Hijji et al.; 71.4 years in Rickard et al.) and 67.07 years for patients who were not replanted (73 years in Döring et al.; 60.7 years in Al-Hijji et al.; 66.4 years in Rickard et al.).

#### Comorbidities

The identified comorbidities were as follows: anticoagulation, chronic kidney disease, DM, HF, heart rhythm disorders, heart organic disease, hypertension, and coronary artery diseases. Anticoagulation was observed in 64% of 581 replanted patients and 55% of 97 patients who were not replanted in Al-Hijji et al. Rickard et al. highlighted an anticoagulation prevalence of 62% among 81 replanted cases and 50% among 60 patients who were not replanted. Al-Hijji et al. reported an HF prevalence of 100% in a sample of 581 replanted patients and 97 patients who were not replanted. Heart organic disease is observed in 24% of 191 replanted patients and 14% of 111 patients who were not replanted as analyzed by Döring et al., and 61% of 581 replanted patients and 33% of 97 patients who were not replanted as studied by Al-Hijji et al.

47.5% of 581 replanted cases and 34% of 97 patients who were not replanted were hypertensive in Al-Hijji et al. Hypertension prevalence was 58% among 81 replanted patients and 52% of 60 patients who were not replanted in Rickard et al. DM, heart rhythm disorders, and coronary artery diseases were described in all three studies. DM was present in 34% of 853 replanted patients (47% of 191 cases in Döring et al.; 29% of 581 patients in Al-Hijji et al.; 38% of 81 cases in Rickard et al.) and 33% of 268 patients who were not replanted (47% of 111 cases in Döring et al.; 7% of 97 patients in Al-Hijji et al.; 48% of 60 cases in Rickard et al.). Sixty-five percent of 853 replanted patients (59% of 191 cases in Döring et al.; 69% of 581 patients in Al-Hijji et al.; 53% of 81 cases in Rickard et al.) and 49% of 268 patients who were not replanted (64% of 111 cases in Döring et al.; 34% of 97 patients in Al-Hijji et al.; 47% of 60 cases in Rickard et al.) suffered from heart rhythm disorders. Coronary artery diseases were observed in 42% of 853 replanted patients (18% of 191 cases in Döring et al.; 46% of 581 cases in Al-Hijji et al.; 73% of 81 patients in Rickard et al.) and 36% of 268 patients who were not replanted (22% of 111 patients in Döring et al.; 37% of 97 patients in Al-Hijji et al.; 60% of 60 cases in Rickard et al.).

We used *t*-test observing significant differences (*p* < 0.05) between the two replanted and no replanted populations concerning anticoagulation therapy (*p* = 0.001), heart failure (*p* = 0.002), chronic kidney disease (*p* = 0.019), organic heart disease (*p* = 0.002), heart rhythm disorders (*p* = 0.012), and hypertension (*p* = 0.024). The replanted population presented more comorbidities than no replanted population.

#### Deaths

Survival rates in the replanted group were higher than in the group of patients who were not replanted in all three studies.

The overall number of deaths was 106 (40% of 268 patients) in the group of patients who were not replanted and 128 (15% of 853 patients) in replanted patients.

We observed significant differences between the two replanted and no replanted populations: the *p*-value was equal to 0.002 (*p* < 0.05; Table 6A), highlighting a higher replanted survival than those who were not replanted.

**Table 6A:** Frequency distribution for survival in replanted and not-replanted patients.

**Dead**							**Survived**						**Study size**	**Percentage**	***p*-value**
	**Replantation**	**Percentage**	**No** ** replantation**	**Percentage**	**All** ** dead**	**Percentage**	**Replantation**	**Percentage**	**No** ** replantation**	**Percentage**	**All** ** survived**	**Percentage**			
Döring et al. (37)	55	18.2%	37	12.3%	92	30.5%	136	45.0%	74	24.5%	210	69.5%	302	100%	0.02
Al-Hijji et al. (38)	41	6.0%	39	5.8%	80	11.8%	540	79.6%	58	8.6%	598	88.2%	678	100%	
Rickard et al. (29)	32	22.7%	30	21.3%	62	44.0%	49	34.8%	30	21.3%	79	56.0%	141	100%	

We applied other statistical methods. In a prospective study by Döring et al., relative risk (RR) allowed measuring an eventual correlation between replantation/no replantation and survival: RR was equal to 0.864. No replantation, after extraction, determined a 15.8% higher mortality rate than replantation. No replantation was not a statistically significant risk (95% confidence interval (RR) = [0.612; 1.220]).

The odds ratio (OR) allowed quantifying the association between replantation/no replantation and survival in retrospective studies (Al-Hijji et al. and Rickard et al.). The application of inconsistent tests highlighted a wide heterogeneity of these studies' population. We applied an unsuccessful random effects model to contain the heterogeneity, deciding to separately analyze the two populations. A model with variable effects did not decrease the heterogeneity (I∧2) that was equal to 93.91% in both models (Tables 6B,C).

**Table 6B:**
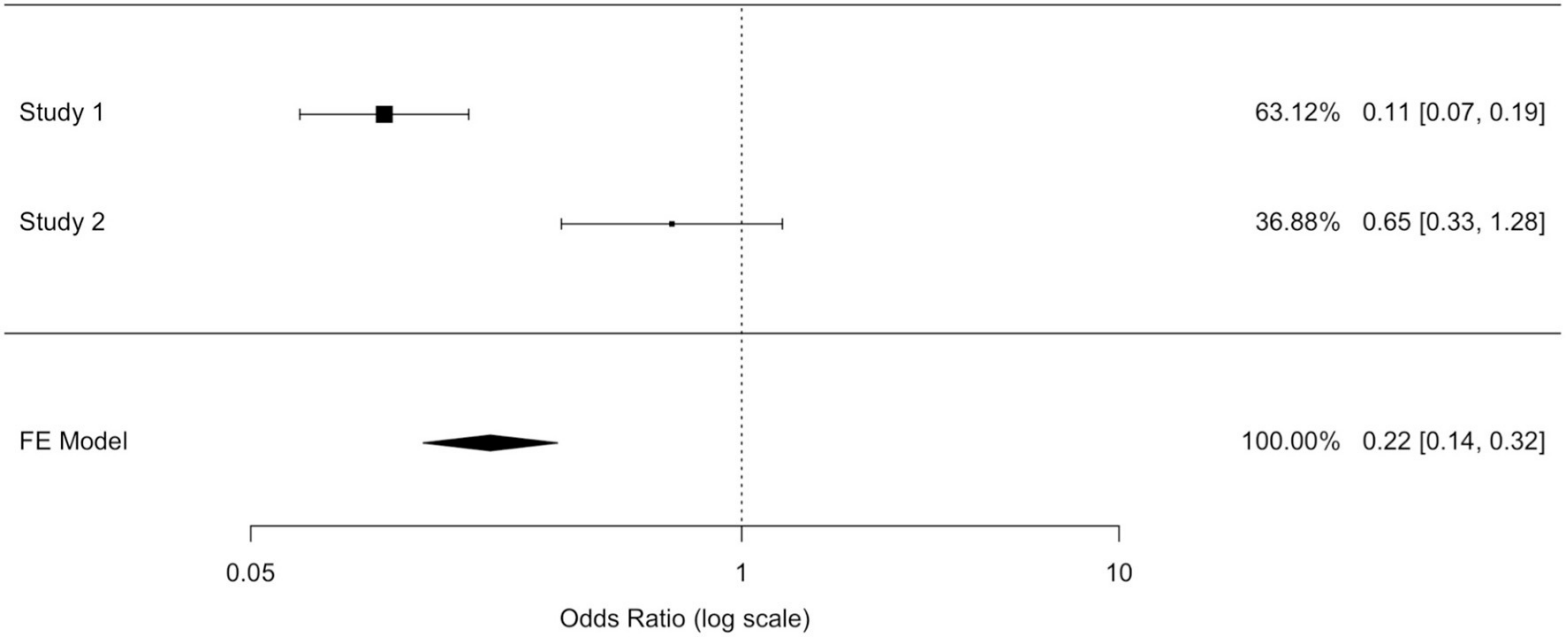
Model with fixed effects.

**Table 6C:**
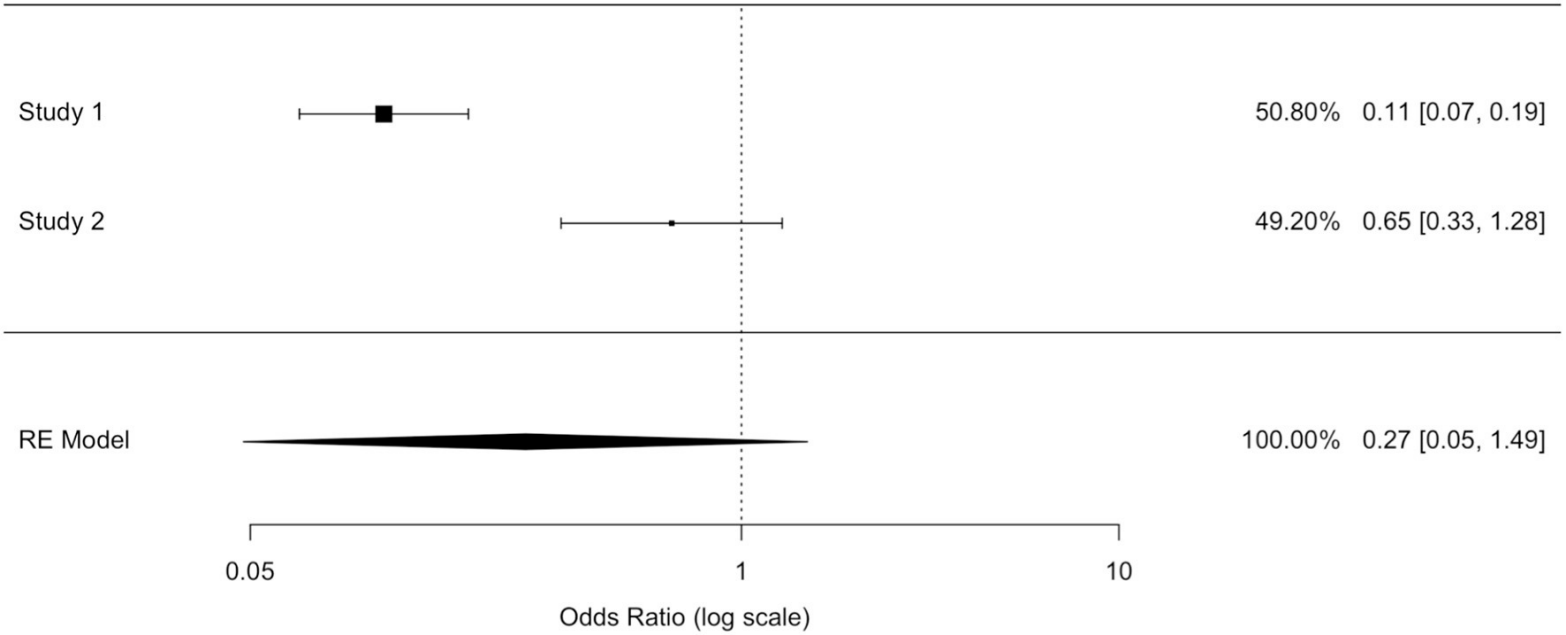
Model with variable effects.

In the study by Al-Hijji et al. (38), where patients who were not replanted had a higher mortality rate than replanted, the OR was equal to 0.113 (where for a simpler reading we performed the following transformation: 1/0.113 = 8.856): not replanted death was 8.856 times more plausible. The OR value reached statistical significance (95% CI (OR) = [0.067; 0.189]); hence, the replanted population showed a survival rate eight times higher than the not replanted group.

In Rickard et al. (29) not replanted death is 1.532 times more plausible: the OR was equal to 0.653 (where for a simpler reading we performed the following transformation: 1/0.653 = 1.532); this value was not statistically significant (95% CI (OR) = [0.333; 1.282]).

Two of the three studies revealed no statistically significant differences in the overall survival between the two populations.

We could not exclude the correlation between replantation and higher survival, although the largest study (38) exhibited a higher and statistically significant survival in the replanted population, and the RR and OR estimated a higher survival in the replantation population.

After ignoring the high value of the inconsistent test, a fixed effects model ensured a more reliable estimate as well as to highlight a protection factor for the replanted population survival. The fixed effects model presented an OR value of 0.22 (for a simpler reading we performed the following transformation: 1/0.22 = 4.54) confirming the replanted population's higher survival. Death was 4.5 times more plausible in patients who were not replanted than those who were replanted. The difference between patients who were replanted and not replanted was statistically significant (IC95% (OR) = [0.14; 0.32]).

### Correlation Between Alteration of Hematological Parameters and Poor Prognosis Analysis

Tang et al. (41) and Papageorgiou et al. (35) analyzed the association between hematological parameters and prognosis in patients who were replanted (489 cases in Tang et al.; 612 cases in Papageorgiou et al.) (Table 7).

**Table 7:** Pre-operative inflammatory markers.

**Articles**		**Papageorgiou et al**. **(****35****)**						
		**NA**		**Alive at follow-up**		**Death/Transplant**		***P*-value**
			**Percentage**		**Percentage**		**Percentage**	
Sample size		33	5.4%	247	40.4%	332	54.2%	
Gender	M			165	66.8%	253	76.2%	0.016
	F			82	33.2%	79	23.8%	
Median age				62.8 ± 13.2		66.9 ± 13.9		0.337
WBC				7.7 ± 2.6 × 10^3^/mm^3^		10.5 ± 4.3 × 10^3^/mm^3^		0.137
Lymphocytes				25 ± 9%		21 ± 10%		0.804
RDW				14.2 ± 1.5%		15.3 ± 2.1%		0.202
Platelet				232 ± 84 × 10^3^/mm^3^		210 ± 71 × 10^3^/mm^3^		0.652
Hemoglobin				12.9 ± 17 g/dL		12.4 ± 19 g/dL		0.515
**Articles**		**Tang et al**. **(****41****)**						
				**Patients with leukocytosis**	**Percentage**	**Patients without leukocytosis**	**Percentage**	***P*****-value**
Sample size				127	26%	362	74%	
Gender	M			99	25.8%	284	74.2%	0.997
	F			28	26.4%	78	73.6%	
Median age				51.21 ± 13.01		56.53 ± 12.96		0.285
WBC				13.20 + 3.56 K/μL		7.38 + 1.65 K/μL		0.002
CRP				5.29 + 6.04 mg/dL		2.80 + 4.05 mg/dL		0.748
Hemoglobin				11.22 + 2.18 g/dL		11.79 + 8.14 g/dL		0.447
N° death				41	32.3%	107	29.6%	0.643
Device infection				26	20.5%	95	26.2%	0.231
Preoperative mortality				8	6.3%	16	4.4%	0.545
RV failure				21	16.5%	17	4.7%	0.028

*M, male; F, female; WBC, white blood cells; RDW, red distribution width; CRP, C-reactive protein; RV, right ventricular*.

#### Age and Gender

A sample of 489 patients was divided into two groups in the Tang et al. study: 127 patients with leukocytosis and 362 patients without leukocytosis. There was a higher male prevalence: 78% in both groups. The median age of patients with leukocytosis was slightly lower than that of patients without leukocytosis (51.21 ± 13.01 years vs. 56.53 ± 12.96 years).

A sample of 612 patients was divided into two groups in Papageorgiou et al. (35): 247 alive at follow-up patients and 332 death/transplant patients. There was a higher male prevalence in both groups: 67% in alive at the follow-up group and 76% in the death/transplant group. The median age was slightly higher in the death/transplant group (66.9 ± 13.9 years) than that is alive at the follow-up group (62.8 ± 13.2 years).

#### Alteration of Hematological Parameters

Tang et al. reported a white blood cell count (WBC) median value of 13.20 ± 3.56 K/μL in patients with leukocytosis and 7.38 + 1.65 K/μL in patients without leukocytosis. The C-reactive protein (CRP) median value was 5.29 ± 6.04 mg/dL in patients with leukocytosis and 2.80 ± 4.05 mg/dL in 362 patients without leukocytosis. Patients with leukocytosis had therefore a higher median value of CRP, an important inflammatory marker. Hemoglobin values were similar in both groups (11.22 ± 2.18 g/dL in patients with leukocytosis vs. 11.79 ± 8.14 g/dL in patients without leukocytosis).

Papageorgiou et al. highlighted a WBC median value in the death/transplant patient group (10.5 ± 4.3) that is higher than that in the alive at follow-up patient group (7.7 ± 2.6).

#### Deaths and Outcomes

Death prevalence in patients with leukocytosis was higher than in patients without leukocytosis (32 vs. 29.5%). The outcome of the two groups was also different: 20% of patients with leukocytosis and 26% of patients without leukocytosis developed CIED infection. No relevant correlation between preoperative leukocytosis and CIED infection was seen. Intraoperative mortality in patients with leukocytosis was higher than in those without leukocytosis (6 vs. 4%). A relevant correlation between leukocytosis and RV failure was observed: development of RV failure was seen in 16.5% of patients with leukocytosis and only in 5% of those without leukocytosis.

Papageorgiou et al. observed a death/transplant rate of 54%. The authors pointed out relevant correlations between high RDW/low platelet value and mortality. The median value of RDW was 15.3% ± 2.1 in the death/transplant group and 14.2% ± 1.5 in the alive at follow-up group. The median value of platelet was 210 ± 71 × 10^3^/mm^3^ in the death/transplant group and 232 ± 84 × 10^3^/mm^3^ in the alive at follow-up group.

Males had a higher probability of death/transplant than females (76.2 vs. 23.8%, *p* = 0.016). Alternatively, we did not detect a significant difference in age between those who survived compared to those who died/were transplanted (*p* = 0.337). Similarly, there were no significant differences in WBC, lymphocytes, RDW, platelets, or hemoglobin.

In Tang et al., there were no statistically significant differences for correlation with survival (OR = 1.136; 95% CI (OR) = [0.735; 1.756]), infection (OR = 0.724; 95% CI (OR) = [0.443; 1.1.82]), and intraoperative mortality (OR = 1.454; 95% CI (OR) = [0.443; 1.1.82]).

Using OR values, some considerations were made. Device infection was 1.382 times more plausible in patients without leukocytosis. These data suggested a protective effect of leukocytosis against device infection, although this value was not statistically significant. Operative mortality was 1.454 times more likely in patients with leukocytosis (OR = 1.454). OR and CI were not particularly reliable data, secondary to low intraoperative mortality. Other studies, on the other hand, showed a statistically significant association between RVF and leukocytosis (OR = 4.021; 95% CI (OR) = [2.046; 7.900]). Patients with leukocytosis had a greater tendency to RVF development, and RVF was four times more plausible in patients with leukocytosis.

We confirmed these data by applying the *t*-test. We did not observe significant differences between the two male and female populations: the *p*-value was equal to 0.997 (*p* > 0.05). Patients with and without leukocytosis showed male and female same distributions. Both groups' median age did not show a significant difference (*p* = 0.285). We compared CRD and RDW and hemoglobin: no statistical differences between the two populations were seen. CRD and hemoglobin values were higher than 0.05, not showing statistical differences. The comparison between the proportions of dead patients did not note significant differences (*p* = 0.643). In addition, we made a comparison between CIED infection mortality and operative mortality in patients with and without leukocytosis: the two groups had same proportion (*p* > 0.05). Finally, we analyzed RVD in the two groups as a post-operative outcome. We highlighted a statistical significant difference regarding RVD development: the *p*-value was equal to 0.028 (*p* < 0.05); patients with leukocytosis presented a higher probability of RVD occurrence.

Papageorgiou et al. was the only study available for mortality analysis according to gender. Male subjects were more frequently affected than women (Table 7): an adverse event (death/transplant) was 1.592 times more plausible in the male population (OR = 1.592; 95% CI (OR) = [1.104; 2.295]). The female gender ensured better survival after extraction.

## Highlights of Statistical Analysis

Male patients appeared to suffer more (70.7%) from CIED infections.

CIED complete removal and antimicrobial therapy was the most prevalent treatment after extraction (99.2% overall prevalence).

Staphylococcal infection was the most common infection type.

In many studies, more than half of the patients received CIED replantation.

Studies showed differences in death causes, time to death, and percentages of deaths; most deaths were in-hospital.

Replantation after extraction ensured a better survival than no replantation, although only one study showed statistical significance.

No correlation was found between patients with/without leukocytosis and gender, survival, device infection, or intraoperative mortality.

We detected a statistically significant association between leukocytosis and RVF: patients with leukocytosis exhibited a higher probability of RVF development.

Female patients revealed a higher survival rate than males.

## Discussion

### Findings of the Study

Due to an improved quality of life and an evolution in the diagnostic and therapeutic approaches, the increase in the average age of population caused a significant increase in heart diseases and CIED implantations. Despite the benefits, implant or replant surgical procedure has a high risk of infection.

We analyzed multiple topics of CIED infections by a thorough literature review in our study.

Epidemiology analysis confirmed staphylococcal prevalence, following literature data (23). They are ubiquitous bacteria in the human skin; their migration to the surgical site is facilitated by surgery. Many studies highlighted the prevalence of *S. aureus* for several years. CoNS occupied the second place; a prevalence of CoNS was observed in our study highlighting a turnaround, in accordance with new emerging data in recent literature (18, 22). Bongiorni et al. reported *S. epidermidis* prevalence among CoNS; this datum might suggest a possible involvement of specific host or CIED factors in CoNS infections: it is supposed that CoNS infect the CIED surface during device insertion (18). Guenther et al. have shown that CoNS may easily evade the immune system than *S. aureus*. CoNS are ubiquitous in the deep layers of the skin; thus, there is a higher risk of sample contamination in CoNS than in *S. aureus* infections. At least two positive blood cultures are required for a definite diagnosis of CoNS bloodstream infection (BSI) (23). An empirical antibiotic therapy against staphylococci is required, considering that staphylococcal infections constitute two-thirds of CIED infections. Staphylococcal methicillin susceptibility may cause empirical therapy changes, such as the introduction of antistaphylococcal penicillins in place of vancomycin (21).

Non-staphylococcal infections represent 10–30% of CIED infections, and they are characterized by the following: a variety of causative microorganism population; an onset of infection later than staphylococcal infections (2 years vs. 3–6 weeks); and a low mortality rate and false positive prevalence, secondary to rare skin contamination (25). Fungal infections (1% of CIED infections) affect mostly immunocompromised patients with a high mortality rate; *Candida* spp. is the most frequent fungus (32).

We also analyzed the characteristics of patients with CIED infections: there was an evident high male prevalence (74% of 11,895 patients examined in all 19 articles); cardiological diseases were the most frequent comorbidities, often representing the CIED implantation cause. Heart failure was the prevalent cardiological comorbidity (46% of 10,242 cases).

Recent studies highlighted a relevant correlation between the alteration of hematological parameters and HF development. Preoperative WBC and CRP high values increased the risk of RVF development. Catecholamine decreases myocardial contractility, inducing a low cardiac output syndrome with consequent tissue ischemia. Leukocytosis can be considered as a tissue hypoperfusion and stress marker. Inflammation constitutes one of the HF-related pathological events (42); various studies (43–45) have underlined the pro-inflammatory markers' (such as tumor necrosis factor α [TNF-α], interleukins IL-6 e IL-8) importance in HF development. These markers may induce myocardial and vascular remodeling with consequent myocardial hypertrophy and fibrosis, recruiting pro-inflammatory cells (46).

Others studies also observed a relevant correlation between the increased RDW value/decreased platelet count and poor prognosis. It has been hypothesized that inflammation may increase the RDW value, inhibiting the erythrocyte maturation and promoting reticulocyte migration into the peripheral circulation (35). Reticulocytes increase the RDW value, inducing hypervolemia that raises vascular resistance and high blood viscosity, which increases ventricular work causing cardiac hypertrophy. Inflammation may also change the coagulation profile. TNF-α induces platelet adhesion, favoring the formation of thrombi and reducing the platelet count. Clogged blood vessels destroy the platelet in the bloodstream, causing the formation of platelet microparticles (PMPs). PMPs induce thrombin and coagulation activation, with formation of thrombi and development of acute or chronic cardiac diseases.

No correlation was observed between preoperative leukocytosis and CIED infection. Therefore, the poor prognosis may be related to a high RDW value and a decreased platelet count; reinfection may be secondary to factors other than leukocytosis.

The high risk of death in case of CIED implantation or replantation delay is known.

In conclusion, a relevant correlation between leukocytosis and RV failure was observed. HF may be related to a high RDW value and a decreased platelet count because of myocardial and vascular remodeling and activation of coagulation cascade. No correlation between preoperative leukocytosis and CIED infection was highlighted. High risk of fatal arrhythmia was pointed out in the case of CIED implantation/replantation delay.

Data concerning the correlation between alteration of hematological parameters and poor prognosis/death were missing in many studies because most operators do not perform implants in patients with signs of infection; implantation or replantation/revision is recommended to be delayed in case of signs of systemic infection (47). Some data suggest that the presence of fever increases the risk of infection. The role of general markers of infection (CRP or white blood cell count) has not been studied. It is preferable to delay the procedure until sepsis has resolved, in case of acute implantation.

Further data about this topic are currently awaited to outline a treatment plan in case of a need for CIED implantation in patients with alteration of hematological parameters.

### Study Limitations

Most studies did not analyze all parameters taken into account in our study. There are currently a few available studies about the correlation between altering hematological parameters and poor prognosis in CIED-implanted patients. Study heterogeneity.

## Data Availability Statement

The raw data supporting the conclusions of this article will be made available by the authors, without undue reservation.

## Author's Note

We conducted a literature research, discussing each article and establishing inclusion and exclusion criteria. We analyzed master data considering new literature trend and on-going debate. Finally, we carried out this study considering the need to outline treatment plan in case of need for CIED implantation in patients with alteration of hematological parameters.

## Author Contributions

GP and CB set the systematic review, selected and analyzed data and wrote the results and discussion. VA processed statistical data and wrote statistical results. AA and SZ reviewed the selected data and wrote discussion and conclusion. PD analyzed cardiological and clinical data. EM and GM contributed to analyze clinical data and completed references. All authors contributed to the article and approved the submitted version.

## Conflict of Interest

The authors declare that the research was conducted in the absence of any commercial or financial relationships that could be construed as a potential conflict of interest.
